# Succession of Bacterial Community During the Initial Aerobic, Intense Fermentation, and Stable Phases of Whole-Plant Corn Silages Treated With Lactic Acid Bacteria Suspensions Prepared From Other Silages

**DOI:** 10.3389/fmicb.2021.655095

**Published:** 2021-03-26

**Authors:** Lin Sun, Chunsheng Bai, Haiwen Xu, Na Na, Yun Jiang, Guomei Yin, Sibo Liu, Yanlin Xue

**Affiliations:** ^1^Inner Mongolia Academy of Agriculture and Animal Husbandry Science, Hohhot, China; ^2^Inner Mongolia Engineering Research Center of Development and Utilization of Microbial Resources in Silage, Hohhot, China; ^3^Horticultural College, Shenyang Agricultural University, Shenyang, China; ^4^College of Foreign Languages, Inner Mongolia University of Finance and Economics, Hohhot, China; ^5^Department of Animal Sciences, University of Florida, Gainesville, FL, United States

**Keywords:** whole-plant corn silage, bacterial community succession, lactic acid bacteria suspension, initial aerobic phase, intense fermentation phase, stable phase, lactic acid bacteria fermentation relay

## Abstract

The present study was aimed at investigating the bacterial community in lactic acid bacteria (LAB) suspensions prepared from whole-plant corn silage (LAB suspension-CS) and *Elymus sibiricus* silage (LAB suspension-ES) and the bacterial community succession of whole-plant corn silages inoculated with LAB suspension-CS or LAB suspension-ES during initial aerobic phase, intense fermentation phase, and stable phase. The LAB suspensions were cultured in sterile Man, Rogosa, Sharpe broth at 37°C for 24 h and used as inoculants for ensiling. The chopped whole-plant corn was treated with distilled water (CK), LAB suspension-CS (CSL), or LAB suspension-ES (ESL) and then ensiled in vacuum-sealed plastic bags containing 500 g of fresh forage. Silages were sampled at 0 h, anaerobic state (A), 3 h, 5 h, 10 h, 24 h, 2 days, 3 days, 10 days, 30 days, and 60 days of ensiling with four replicates for each treatment. The results showed that *Lactobacillus*, *Weissella*, and *Lachnoclostridium*_5 dominated the bacterial community in LAB suspension-CS; *Lactobacillus* was the most predominant bacterial genus in LAB suspension-ES. During the initial aerobic phase (from 0 h to A) of whole-plant corn silage, the pH and the abundances of *Pantoea*, *Klebsiella*, *Rahnella*, *Erwinia*, and *Serratia* increased. During the intense fermentation phase (from A to 3 days), the pH decreased rapidly, and the microbial counts increased exponentially; the most predominant bacterial genus shifted from *Pantoea* to *Weissella*, and then to *Lactobacillus*; inoculating LAB suspensions promoted the bacterial succession and the fermentation process, and LAB suspension-CS was more effective than LAB suspension-ES. During the stable phase (from 3 to 60 days), the pH and the microbial counts decreased, and *Lactobacillus* dominated the bacterial community with a little decrease. The results also confirmed the existence of LAB fermentation relay during fermentation process, which was reflected by *Weissella*, *Lactococcus*, and *Leuconostoc* in the first 5 h; *Weissella*, *Lactococcus*, *Leuconostoc*, *Lactobacillus*, and *Pediococcus* between 5 and 24 h; and *Lactobacillus* from 24 h to 60 days.

## Introduction

Ensiling is an effective method for preserving moist forage crops and supplying quality forage to livestock throughout the year (Guan et al., 2018; Zhang et al., 2019). During the fermentation process, water-soluble carbohydrates are converted into organic acids by lactic acid bacteria (LAB) under anaerobic conditions to reduce pH and inhibit undesirable microorganisms for long-term preservation of silage (Keshri et al., 2018). Generally, the main LAB genera that play a major role in silages include *Lactobacillus*, *Weissella*, *Pediococcus*, *Lactococcus*, *Enterococcus*, and *Leuconostoc* (McGarvey et al., 2013; Muck, 2013; Gharechahi et al., 2017). Additionally, the ensiling fermentation process is highly complex involving many types of microorganisms (Xu et al., 2019), and the process can be divided into four main phases including the initial aerobic phase, intense fermentation phase, stable phase, and aerobic feed-out phase (Weinberg and Muck, 1996; Dunière et al., 2013; Ávila and Carvalho, 2019). Therefore, understanding the succession of microorganisms and the correlation between microorganisms and fermentation quality in different phases of fermentation may reveal the fermentation process and provide a scientific basis for modulation of silage fermentation.

Whole-plant corn silage is the most common forage for ruminant worldwide because of the good fermentation quality and high nutritional value (Khan et al., 2015; Zhang et al., 2019). In the past decade, the microbial communities during ensiling have become a research focus of silages with the development of next-generation sequencing technologies (Romero et al., 2018). Gharechahi et al. (2017), Keshri et al. (2018), and Xu et al. (2020) reported the dynamics of microbial community during the ensiling of whole-plant corn silages treated with LAB or collected from different locations. Guan et al. (2018) determined the bacterial community in corn silages prepared with farm bunker-silo in Southwest China. Other previous studies revealed the bacterial community in whole-plant corn silages inoculated with LAB (Xu et al., 2019; Zhang et al., 2019), and the bacterial and fungal communities in whole-plant corn silages after 5 days of aerobic exposure (Keshri et al., 2018). However, little is known regarding the successions of microbial community during the initial aerobic phase and the fermentation relay of LAB during entire fermentation of whole-plant corn silages.

The LAB was usually used as silage additives to promote the ensiling process and improve the fermentation quality of end-silages (Weinberg and Muck, 1996). Some recent studies indicated that adding inoculants (containing selected LAB strains) changed the microbial community by making LAB dominated microorganism as soon as possible (Keshri et al., 2018; Guan et al., 2020; Xu et al., 2020). Several authors have reported that LAB prepared from alfalfa is more effective in improving the fermentation quality of alfalfa silage than that from other forage sources (Ohshima et al., 1997; Wang et al., 2009; Denek et al., 2011). Moreover, Ali et al. (2020) found that inoculating epiphytic microbiota from red clover has a greater effect on improving microbial succession and fermentation quality of red clover silages than that from maize and sorghum. To the best of our knowledge, no studies have reported the microbial succession of whole-plant corn silages ensiled with LAB prepared from whole-plant corn silages and other silages. *Elymus sibiricus* is a tall-growing and perennial bunchgrass widely distributed in Europe, Asia, and North America (Klebesadel, 1969), and used as forage because of high yield potential and good quality (Wang et al., 2017). Moreover, *E. sibiricus* has become a grass for artificial grassland and been ensiled for ruminants in China (Sun et al., 2009; Li et al., 2016).

We hypothesized that inoculation of the LAB suspensions prepared from whole crop corn and *E. sibiricus silages* at ensiling may alter the bacterial community and their successions in whole crop corn silage. Thus, the objectives of this study were to determine the bacterial community in LAB suspensions prepared from whole-plant corn silage and *E. sibiricus* silage and to characterize the changes in the bacterial community during the initial aerobic phase, intense fermentation phase, and stable phase of the anaerobic fermentation process in whole-plant corn silage inoculated with two different LAB suspensions prepared from whole crop corn and *E. sibiricus* silages.

## Materials and Methods

### Preparing LAB Suspension

The silage samples (about 500 g) for preparing LAB suspensions were collected from wrapped whole-plant corn silage (four randomly selected bales; density >700 kg/m^3^) and wrapped *E. sibiricus* silage (four randomly selected bales; density >700 kg/m^3^), respectively, on September 5, 2019. The wrapped silages were ensiled for about 350 days on an experimental farm of Inner Mongolia Academy of Agriculture and Animal Husbandry Science, Hohhot, China. The samples were separately packed in a plastic bag (food grade, 300 mm × 400 mm; Qingye, Beijing, China) by a vacuum sealer (DZ-300; Qingye, Beijing, China), and then transferred to the laboratory in an ice box. The silages (20 g) were individually mixed uniformly with 180 ml of sterile Man, Rogosa, Sharpe (MRS) broth in a reagent bottle (200 ml) by a shaker (HY-150, Wuhan Huicheng Biological Technology Co., Ltd., Wuhan, China) at 4°C for 30 min and then cultured at 37°C in an incubator (LRH-70, Shanghai Yiheng Scientific Instruments Co., Ltd., Shanghai, China) for 24 h. The 20 ml of LAB suspensions was drawn from each reagent bottle, placed into a sterilized centrifuge tube, and then stored at −80°C for analyzing bacterial community. The pH and LAB count of the silage samples and the LAB suspensions were analyzed. The LAB suspensions were stored at 4°C and used as an inoculant for ensiling whole-plant corn in 5 days.

### Preparing Silages and Sampling

Corn (*Zea mays* L.) was grown on the same farm. Whole corn plants were harvested at the two-thirds milk-line stage from four different fields as replicates on September 10, 2019. The fresh forages from each field were separately chopped into 1- to 2-cm pieces, mixed thoroughly, and then divided into three batches of film for three treatments as follows: CK, spraying 3.0 ml/kg fresh weight (FW) of distilled water; CSL, spraying 3.0 ml/kg FW of LAB suspension made from whole-plant corn silage (LAB suspension-CS); and ESL, spraying with a mixture of 1.0 ml/kg FW of LAB suspension made from *E. sibiricus* silage (LAB suspension-ES) and 2.0 ml/kg (FW) distilled water. After mixing uniformly, approximately 500 g of forage was packed into a plastic bag and sealed with a vacuum sealer; 44 bags of silage were prepared per treatment (11 bags per field), and a portable oxygen meter (KP810, Henan Zhong an Electronic Detection Technology Co., Ltd., Zhengzhou, China) was sealed in a bag per treatment of each field for detecting oxygen content. The silages were stored in laboratory and sampled after ensiling for 0 h, anaerobic state (A), 3 h, 5 h, 10 h, 24 h, 2 days, 3 days, 10 days, 30 days, and 60 days. When the oxygen content in silages reduced to 0, the silage was in anaerobic state. The 20 g of sample from each bag was placed into a self-styled bag and stored at −80°C for analyzing bacterial community.

### Analysis

The silages were dried in a forced-air oven (BPG-9240A, Shanghai Yiheng Scientific Instrument Co., Ltd., Shanghai, China) at 65°C for 48 h, ground through a 1-mm screen using a mill (FS-6D; Fichi Machinery Equipment Co., Ltd., Shandong, China) and then dried at 105°C until reaching a constant mass for detecting dry matter content. The mixture of 25 g fresh silage and 225 ml sterile water was homogenized for 100 s in a flap-type sterile homogenizer (JX-05, Shanghai Jingxin Industrial Development Co., Ltd., Shanghai, China) and filtered through four layers of cheesecloth to prepare silage extracts. The pH of silage was measured by pH meter; the counts of LAB, Coliforms, bacteria, and yeast were determined by culturing on MRS agar, violet red bile agar, nutrient agar, and potato dextrose agar, respectively, at 37°C for 48 h (Cai, 1999).

The bacterial community of the LAB suspensions and the whole-plant corn silages were analyzed. The DNA extraction was operated using the E.Z.N.A. ^®^Stool DNA Kit (D4015, Omega, Inc., United States) according to the manufacturer’s instructions. The V3–V4 region of the bacterial rRNA gene was amplified by polymerase chain reaction (PCR) (98°C for 30 s followed by 32 cycles of denaturation at 98°C for 10 s, annealing at 54°C for 30 s, and extension at 72°C for 45 s and a final extension at 72°C for 10 min) with primers 341F (5′-CCTACGGGNGGCWGCAG-3′) and 805R (5′-GACTACHVGGGTATCTAATCC-3′) (Logue et al., 2016). The PCR products were purified by AMPure XT beads (Beckman Coulter Genomics, Danvers, MA, United States) and quantified by Qubit (Invitrogen, United States). The samples were sequenced on an Illumina NovaSeq PE250 platform according to the manufacturer’s recommendations. Paired-end reads were assigned to samples based on their unique barcode and truncated by cutting off the barcode and primer sequence. Paired-end reads were merged using FLASH. Quality filtering on the raw reads was performed under specific filtering conditions to obtain the high-quality clean tags according to the fqtrim (v0.94). Chimeric sequences were filtered using Vsearch software (v2.3.4). After dereplication using DADA2, the feature table and feature sequence were obtained. Alpha diversity and beta diversity were calculated by QIIME2, the sequence alignment of species annotation was performed by BLAST, and the alignment database was SILVA and NT-16S. The sequencing data were submitted to the NCBI Sequence Read Archive database (accession number: PRJNA693042). The stacked bars of bacterial genera were made by Excel (Microsoft 365, Microsoft Corporation, Seattle, WA, United States) according to the relative abundance of the bacterial community. The difference in the bacterial community among treatments or ensiling time was analyzed using the Mann–Whitney *U* test and the Kruskal–Wallis test by R version 3.6.1.

### Statistical Analyses

The LAB Count and pH of LAB suspension whole-plant corn silage, and *E. sibiricus* silage were analyzed using GLIM procedure of SAS (version 9.1.3; SAS Inst. Inc., Cary, NC, United States). For the ensiling experiments, data on dry matter, pH, and microbial counts of whole-plant corn silage were analyzed as 3 × 2 factorial design, 3 × 7 factorial design, and 3 × 4 factorial design during the initial aerobic phase, the intense fermentation phase, and the stable phase, respectively. The model included effects of additives, storage time, and their interaction. Differences among additives, and among storage times, were analyzed with the GLIM procedure of SAS. The interaction of additives and storage time was analyzed using the PDIFF procedure of SAS. The correlation between bacterial genera and pH were performed by R version 3.6.1 using the OmicStudio tools at https://www.omicstudio.cn/tool.

## Results

### Characteristic of LAB Suspension

Whole-plant corn silages for preparing LAB suspension had lower pH and less LAB count than *E. sibiricus* silages (*P* < 0.05). Compared to LAB suspension-ES, the LAB suspension-CS had higher pH and lower LAB count (*P* < 0.05) (Table 1).

**TABLE 1:** pH and lactic acid bacteria (LAB) counts of whole-plant corn silage and *Elymus sibiricus* silage (log_10_ colony-forming units/g fresh weight), and of LAB suspension (log_10_ colony-forming units/ml) prepared from the two silages.

Items		Whole-plant corn silage	*Elymus sibiricus* silage	SEM	*P* value
Silages	pH	3.51b	4.71a	0.0128	<0.0001
	LAB	6.05b	7.59a	0.0398	<0.0001
LAB suspension	pH	4.55a	3.76b	0.0176	<0.0001
	LAB	8.49b	9.02a	0.0251	<0.0001

*Values with different lowercase letters show significant differences (*P* < 0.05). SEM, standard error of means.*

**FIGURE 1:**
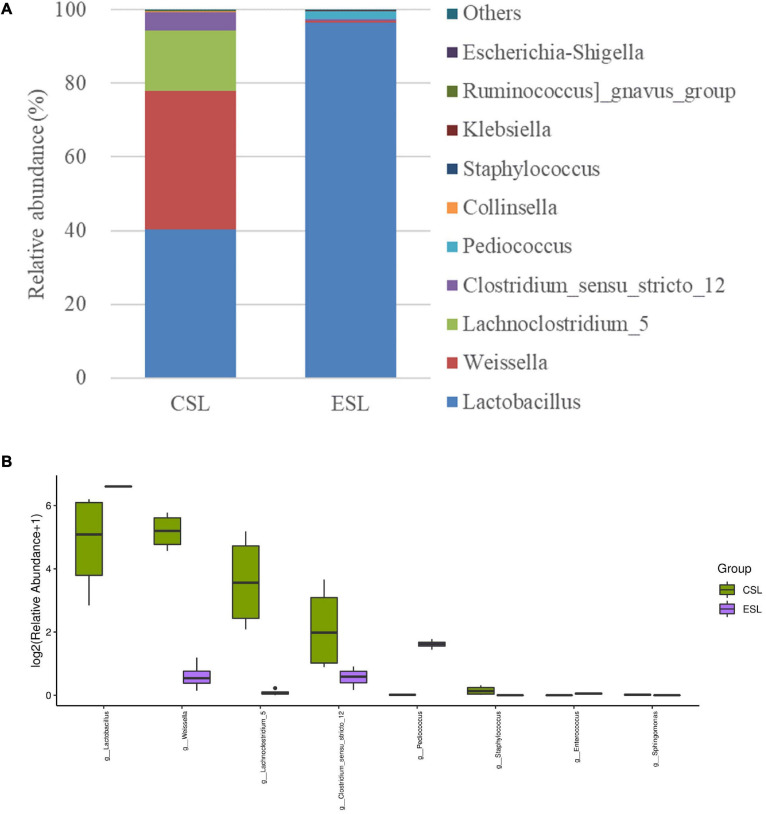
Relative abundance of bacterial communities (genus level) in lactic acid bacterial suspensions prepared from whole-plant corn silage (LAB suspension-CS) and *Elymus sibiricus* silage (LAB suspension-ES) **(A)** and difference in bacterial communities (genus level) between LAB suspension-CS and LAB suspension-ES **(B)**.

The dominant genera in LAB suspension-CS were *Lactobacillus*, *Weissella*, *Lachnoclostridium*_5, and *Clostridium*_sensu_stricto_12 with abundances of 40.3, 37.6, 16.6, and 5.01%, respectively. However, *Lactobacillus* was the most predominant genus in LAB suspension-ES (96.4%), followed by *Pediococcus* and *Clostridium*_sensu_stricto_12 with abundances of 2.07 and 0.50%, respectively (Figure 1A). Additionally, LAB suspension-CS contained greater *Weissella*, *Lachnoclostridium*_5, *Clostridium*_sensu_stricto_12, and *Staphylococcus*, and lower *Lactobacillus* and *Pediococcus* than LAB suspension-ES (*P* < 0.05) (Figure 1B).

### Initial Aerobic Phase

**FIGURE 2:**
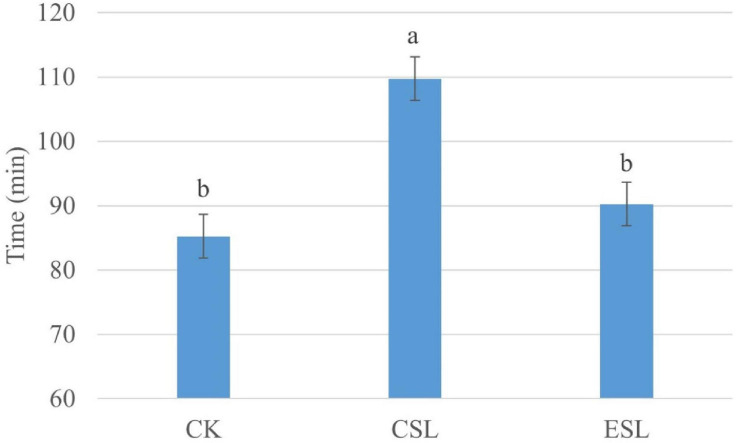
Initial aerobic phase time (min) of whole-plant corn silages. CK, whole-plant corn silage without any lactic acid bacterial suspensions; CSL, whole-plant corn silage treated with lactic acid bacterial suspensions prepared from whole-plant corn silage; ESL, whole-plant corn silage treated with lactic acid bacterial suspensions prepared from *Elymus sibiricus* silage. Values with different lowercase letters show significant differences (*P* < 0.05).

The CSL_0 h and ESL_0 h had lower pH and greater counts of LAB and coliforms than CK_0 h (*P* < 0.05); additionally, ESL_A contained lower pH and higher coliform count than CK_A (*P* < 0.05). The CK_A had higher pH and LAB count than CK_0 h (*P* < 0.05), and CSL_A had greater pH than CSL_0 h (*P* < 0.05) (Table 2). The initial aerobic phase time of CSL was longer than that of CK and ESL (*P* < 0.05) (Figure 2). There was no interactive effect detected between storage time and additives.

**TABLE 2:** Dry matter (DM, g/kg) and microbial counts (log_10_ colony-forming units/g fresh weight) of whole-plant corn silage during the initial aerobic phase.

Items		CK	CSL	ESL	SEM	*P* value
DM	0 h	389	364	378	6.2533	0.0612
	A	385	369	372	5.1200	0.1141
	SEM	6.4928	6.8648	2.9492		
	*P* value	0.6972	0.6593	0.1847		
pH	0 h	6.05Ab	5.88Bb	5.89B	0.0263	0.0022
	A	6.14Aa	6.03ABa	5.94B	0.0340	0.0080
	SEM	0.0173	0.0291	0.0404		
	*P* value	0.0150	0.0115	0.4851		
Lactic acid bacteria	0 h	6.19bB	6.83A	6.93A	0.1939	0.0473
	A	6.59a	6.68	6.55	0.1023	0.6735
	SEM	0.0988	0.0837	0.2353		
	*P* value	0.0267	0.2385	0.2884		
Yeasts	0 h	6.73	7.04	7.15	0.2096	0.3818
	A	6.90	7.05	6.90	0.1098	0.5425
	SEM	0.1461	0.1590	0.1933		
	*P* value	0.4295	0.9575	0.3957		
Coliforms	0 h	7.20B	7.53A	7.47A	0.0669	0.0174
	A	7.22B	7.42AB	7.53A	0.0751	0.0504
	SEM	0.0763	0.0518	0.0816		
	*P* value	0.8766	0.1926	0.6219		
Bacteria	0 h	7.56	8.32	7.88	0.2032	0.0770
	A	7.61	8.28	7.86	0.2377	0.1853
	SEM	0.0454	0.3770	0.0499		
	*P* value	0.5098	0.9498	0.7605		

*Values with different lowercase letters (a and b) indicate significant differences among treatments on the same time. Values with different uppercase letters (A and B) indicate significant differences between 0 h and anaerobic state (A). CK, whole-plant corn silage without any lactic acid bacterial suspensions; CSL, whole-plant corn silage treated with lactic acid bacterial suspensions prepared from whole-plant corn silage; ESL, whole-plant corn silage treated with lactic acid bacterial suspensions prepared from *Elymus sibiricus* silage. A, anaerobic state. SEM, standard error of means.*

**FIGURE 3:**
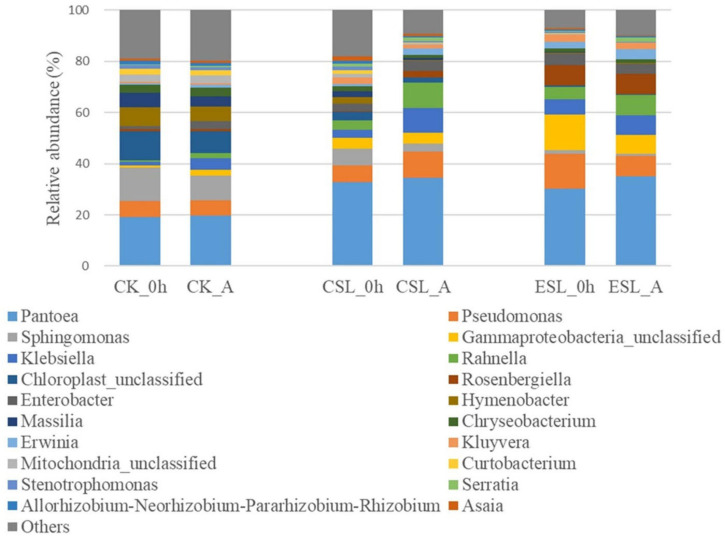
Relative abundance of bacterial communities (genus level) in whole-plant corn silage before ensiling (0 h) and at anaerobic state (A). CK, whole-plant corn silage without any lactic acid bacterial suspensions; CSL, whole-plant corn silage treated with lactic acid bacterial suspensions prepared from whole-plant corn silage; ESL, whole-plant corn silage treated with lactic acid bacterial suspensions prepared from *Elymus sibiricus* silage.

**FIGURE 4:**
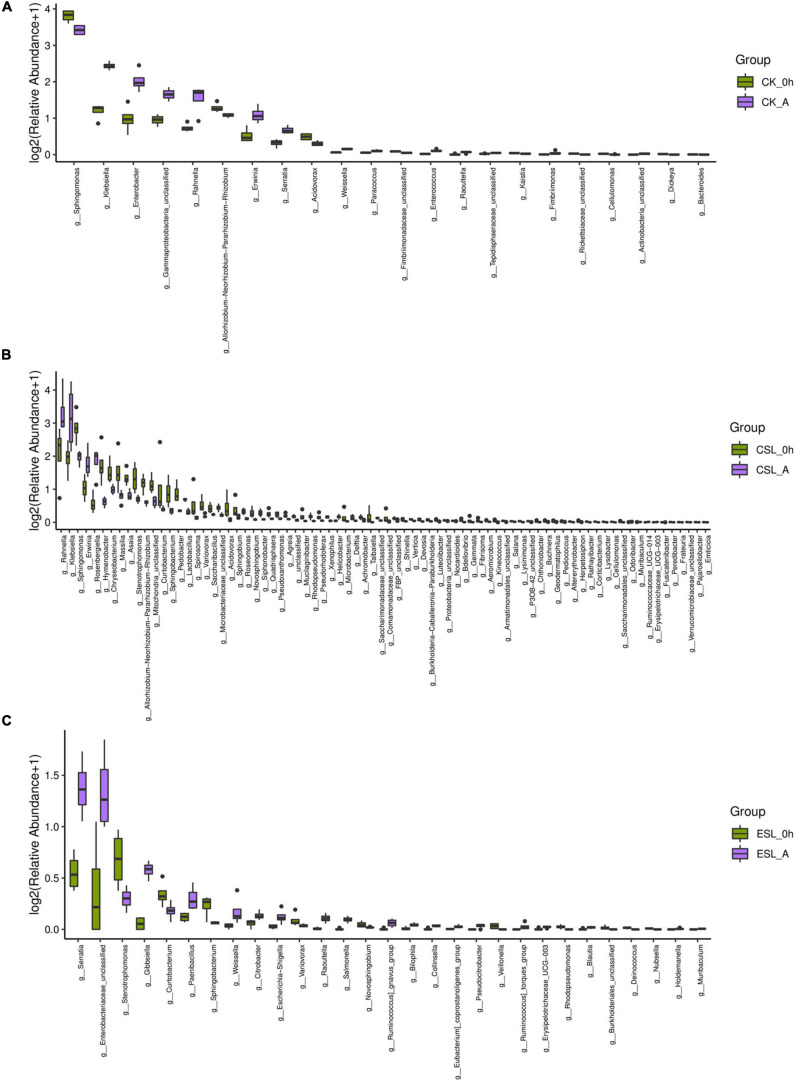
Difference in bacterial communities (genus level) between before ensiling (0 h) and at anaerobic state (A) in whole-plant corn silages without any lactic acid bacterial suspensions (CK, **A**), and treated with lactic acid bacterial suspensions prepared from whole-plant corn silage (CSL, **B**) and from *Elymus sibiricus* silage (ESL, **C**).

During this phase, the predominant bacterial genera in CK, CSL, and ESL were *Pantoea*, *Pseudomonas*, *Sphingomonas*, unclassified *Gammaproteobacteria*, *Klebsiella*, *Rahnella*, *Enterobacter*, and *Chryseobacterium* with more than 1% of abundance (Figure 3). CK_A contained greater *Klebsiella*, *Enterobacter*, *Rahnella*, unclassified *Gammaproteobacteria*, *Serratia*, and *Erwinia*, and lower *Sphingomonas* and *Allorhizobium-Neorhizobium-Pararhizobium-Rhizobium* than CK_0 h (*P* < 0.05). CSL_A contained higher *Rahnella*, *Rosenbergiella*, *Klebsiella*, and *Erwinia*, and lower *Sphingomonas*, *Chryseobacterium*, *Hymenobacter*, *Asaia*, *Stenotrophomonas*, unclassified *Mitochondria*, *Allorhizobium-Neorhizobium-Pararhizobium-Rhizobium*, *Massilia*, and *Curtobacterium* than CSL_0 h (*P* < 0.05); ESL_A contained higher *Serratia* and lower *Stenotrophomonas* and *Curtobacterium* than ESL_0 h (*P* < 0.05) (Figure 4). The abundance of *Weissella* in CK_A and ESL_A was greater than in CK_0 h and ESL_0 h, respectively (*P* < 0.05); however, *Lactobacillus* and *Pediococcus* decreased in CSL during this phase (*P* < 0.05) (Figure 4).

### Intense Fermentation Phase

During this phase, the pH and the coliform count decreased (*P* < 0.05), while the counts of LAB, bacteria, and yeast increased (*P* < 0.05). The ESL had lower pH at A and 10 h, and higher pH at 3 h than CK and CSL (*P* < 0.05). Comparing with CK, the CSL and ESL had lower pH after 24 h (*P* < 0.05), and higher LAB count from 3 h to 2 days (*P* < 0.05). The storage time impacted pH and counts of LAB, coliforms, bacteria, and yeast (*P* < 0.05); the additives (LAB suspension) affected pH and counts of LAB, bacteria, and yeast (*P* < 0.05), which were also interactively influenced by storage time and additives (*P* < 0.05) (Table 3).

**TABLE 3:** Dry matter (DM, g/kg) and microbial counts (log_10_ colony-forming units/g fresh weight) of whole-plant corn silage during the intense fermentation phase.

Items		CK	CSL	ESL	SEM	*P* value	Interaction		
							T	A	T × A
DM	A	385	369ab	372b	5.1200	0.1141		***	*
	3 h	386	375a	379ab	6.1616	0.4881			
	5 h	392A	359abB	377abA	4.8634	0.0035			
	10 h	388A	348bB	380abA	3.3344	<0.0001			
	24 h	391A	365abB	391aA	4.6015	0.0048			
	2 days	379	374a	375ab	3.5375	0.5541			
	3 days	382	371ab	379ab	4.6533	0.2643			
	SEM	4.2870	5.8195	3.7257					
	*P* value	0.3695	0.0420	0.0573					
pH	A	6.14aA	6.03aAB	5.94bB	0.0340	0.0080	***	***	***
	3 h	5.95bB	5.94bB	6.02aA	0.0129	0.0036			
	5 h	5.58c	5.63c	5.53c	0.0235	0.0550			
	10 h	5.11dA	5.10dA	4.89dB	0.0463	0.0135			
	24 h	4.63eA	4.53eB	4.58eAB	0.0178	0.0137			
	2 d	4.25fA	4.04fC	4.14fB	0.0090	<0.0001			
	3 d	4.01gA	3.83gB	3.86gB	0.0109	<0.0001			
	SEM	0.0243	0.0275	0.0244					
	*P* value	<0.0001	<0.0001	<0.0001					
Lactic acid bacteria	A	6.59d	6.68d	6.55d	0.1023	0.6735	***	***	***
	3 h	6.78dB	7.24cA	7.30cA	0.1039	0.0123			
	5 h	8.35cB	8.76bA	9.00bA	0.0924	0.0024			
	10 h	8.70bB	9.37aA	9.41aA	0.0530	<0.0001			
	24 h	9.24aB	9.44aA	9.57aA	0.0488	0.0028			
	2 days	9.26aB	9.41aA	9.44aA	0.0454	0.0448			
	3 days	9.26a	9.52a	9.46a	0.0700	0.0597			
	SEM	0.0744	0.0797	0.0780					
	*P* value	<0.0001	<0.0001	<0.0001					
Yeast	A	6.90f	7.05d	6.90d	0.1098	0.5425	***	***	***
	3 h	6.84f	7.00d	6.78d	0.0920	0.2630			
	5 h	7.17eB	7.77cA	7.38cB	0.0996	0.0065			
	10 h	7.63dB	8.24bA	8.15bA	0.0734	0.0005			
	24 h	8.10cC	8.27bB	8.42aA	0.0444	0.0022			
	2 days	8.36bB	8.42bA	8.43aA	0.0120	0.0053			
	3 days	8.62aC	8.78aA	8.68aB	0.0097	<0.0001			
	SEM	0.0776	0.0659	0.0770					
	*P* value	<0.0001	<0.0001	<0.0001					
Coliforms	A	7.22a	7.42a	7.53a	0.0751	0.0504	***		
	3 h	7.54a	7.37a	7.64a	0.1017	0.2256			
	5 h	7.39a	7.49a	7.61a	0.0775	0.1830			
	10 h	7.71a	7.73a	7.87a	0.0628	0.1986			
	24 h	7.65aA	7.42aB	7.69aA	0.0603	0.0223			
	2 days	5.77bA	4.31bB	5.79bA	0.0954	<0.0001			
	3 days	3.23c	3.37b	2.67c	0.7648	0.7929			
	SEM	0.1781	0.4451	0.1935					
	*P* value	<0.0001	<0.0001	<0.0001					
Bacteria	A	7.61f	8.28c	7.86e	0.2377	0.1853	***	***	***
	3 h	7.70ef	7.80d	7.84e	0.0526	0.2051			
	5 h	7.88eB	8.70bcA	8.77dA	0.0614	<0.0001			
	10 h	8.27dB	8.91abA	8.97cA	0.0285	<0.0001			
	24 h	8.94c	9.14ab	9.21b	0.0920	0.1559			
	2 days	9.19bB	9.40aA	9.47aA	0.0524	0.0112			
	3 days	9.39a	9.45a	9.51a	0.0796	0.5857			
	SEM	0.0639	0.1621	0.0673					
	*P* value	<0.0001	<0.0001	<0.0001					

Values with different lowercase letters (a, b,… g) indicate significant differences among anaerobic state, 3 h, 5 h, 10 h, 24 h, 2 days, and 3 days after ensiling of each treatment. Values with different uppercase letters (A, B, and C) indicate significant differences among treatments on the same time during the intense fermentation phase. CK, whole-plant corn silage without any lactic acid bacterial suspensions; CSL, whole-plant corn silage treated with lactic acid bacterial suspensions prepared from whole-plant corn silage; ESL, whole-plant corn silage treated with lactic acid bacterial suspensions prepared from *Elymus sibiricus* silage. A, anaerobic state. SEM, standard error of means. *, P < 0.05; ***, P < 0.001.

**FIGURE 5:**
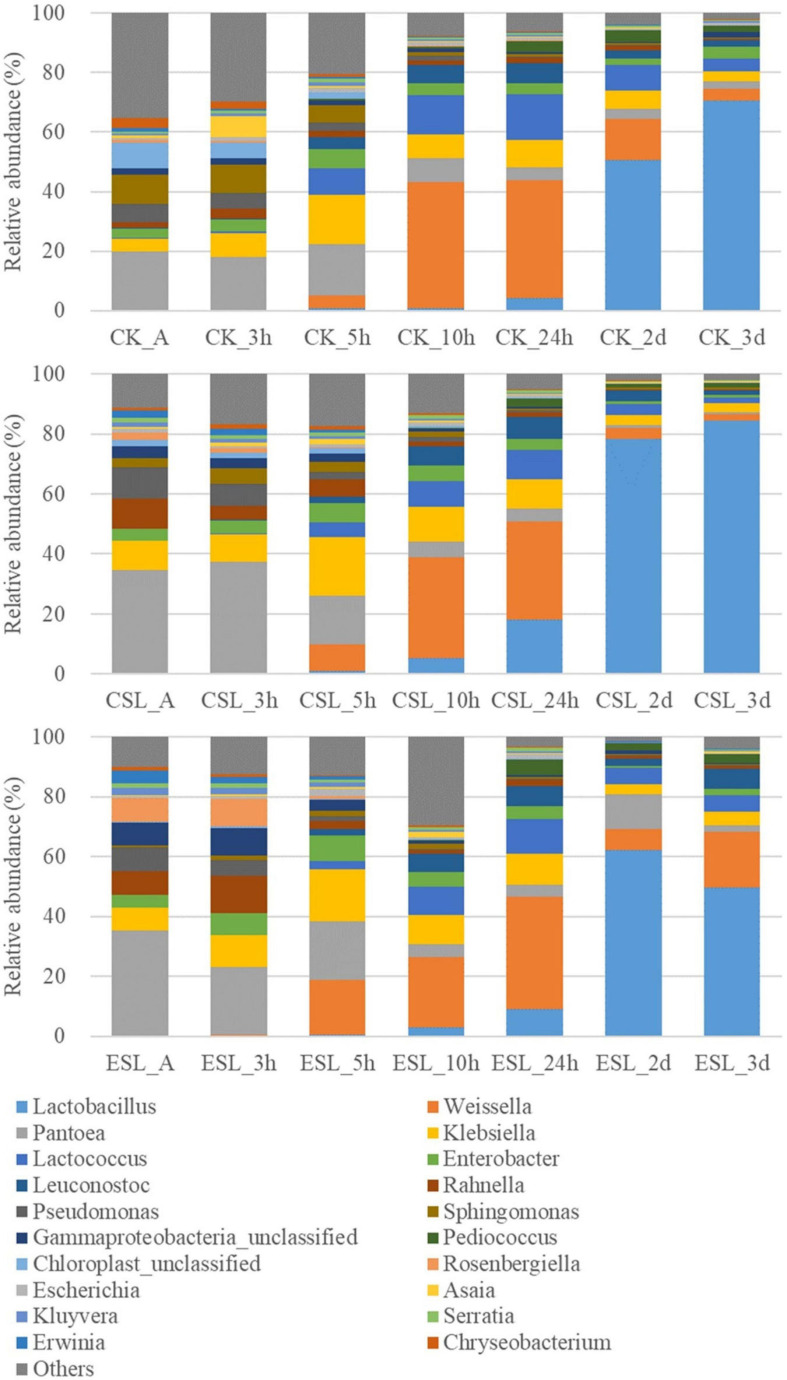
Relative abundance of bacterial communities (genus level) in whole-plant corn silages during the intense fermentation phase. CK, whole-plant corn silage without any lactic acid bacterial suspensions; CSL, whole-plant corn silage treated with lactic acid bacterial suspensions prepared from whole-plant corn silage; ESL, whole-plant corn silage treated with lactic acid bacterial suspensions prepared from *Elymus sibiricus* silage; A, anaerobic state.

During this phase, the abundance of *Lactobacillus* in CK and CSL increased to 70.5 and 84.5% at 3 days, respectively; however, *Weissella* increased to 42.4 and 33.8% at 10 h and then decreased to 4.05 and 2.10% at 3 days, respectively. Additionally, *Lactococcus* and *Leuconostoc* in CK and CSL went up in the first 24 h and then turned down. *Pediococcus* in CK increased to 3.94% at 2 days and then decreased to 2.28% at 3 days. *Pediococcus* in CSL increased to 2.85% at 24 h and then reduced to 1.34% at 3 days. In the ESL, *Lactobacillus* increased to 62.3% at 2 days and then decreased to 49.9% at 3 days; moreover, *Weissella*, *Lactococcus*, *Leuconostoc*, and *Pediococcus* went up in the first 24 h and down at 2 days, and then went up a little bit at 3 days. *Pantoea* in CK and ESL went down during this phage, while in CSL, it increased to 37.0% at 3 h and then went down to 0.67% at 3 days. Moreover, *Klebsiella* and *Enterobacter* in CK, CSL, and ESL went up in the first 5 h and then turned down (Figure 5).

### Stable Phase

During this phase, the pH and the counts of LAB, yeast, coliforms, and bacteria decreased in CK, CSL, and ESL (*P* < 0.05). The CSL and ESL had lower pH than CK, and ESL contained more yeast count than CK and CSL (*P* < 0.05). The storage time and the additives had an effect on pH (*P* < 0.05) (Table 4).

**TABLE 4:** Dry matter (DM, g/kg) and microbial counts (log_10_ colony-forming units/g fresh weight) of whole-plant corn silage during the stable phase.

Items		CK	CSL	ESL	SEM	*P* value	Interaction		
							T	A	T × A
DM	3 days	382ab	371	379	4.6533	0.2643		**	**
	10 days	373b	379	381	3.4621	0.3005			
	30 days	387a	378	380	2.8565	0.0992			
	60 days	388aA	369B	389A	3.9423	0.0106			
	SEM	3.3796	4.0575	3.8884					
	*P* value	0.0354	0.2538	0.2715					
pH	3 days	4.01aA	3.83bB	3.86aB	0.0107	<0.0001	***	***	***
	10 days	3.85b	3.82b	3.83a	0.0082	0.0550			
	30 days	3.90bA	3.87aB	3.86aB	0.0067	0.0136			
	60 days	3.65c	3.58c	3.58b	0.0206	0.0725			
	SEM	0.0149	0.0103	0.0128					
	*P* value	<0.0001	<0.0001	<0.0001					
Lactic acid bacteria	3 days	9.26a	9.52a	9.46a	0.0699	0.0597	***		
	10 days	8.71b	8.99b	8.94b	0.1188	0.2561			
	30 days	8.30c	8.17c	8.20c	0.0675	0.4381			
	60 days	7.68d	7.64d	7.70d	0.0321	0.4029			
	SEM	0.1040	0.0463	0.0741					
	*P* value	<0.0001	<0.0001	<0.0001					
Yeast	3 days	8.62aC	8.78aA	8.68aB	0.0097	<0.0001	***		
	10 days	8.33bB	8.37bB	8.47aA	0.0173	0.0007			
	30 days	8.03c	7.95c	7.99b	0.1116	0.8877			
	60 days	7.23d	7.21d	7.19c	0.0982	0.9544			
	SEM	0.0601	0.0799	0.0829					
	*P* value	<0.0001	<0.0001	<0.0001					
Coliforms	3 days	3.23a	3.37a	2.67a	0.7648	0.7929	***		
	10 days	ND	ND	ND	–	–			
	30 days	ND	ND	ND	–	–			
	60 days	ND	ND	ND	–	–			
	SEM	0.2101	0.5817	0.2369					
	*P* value	<0.0001	0.0028	<0.0001					
Bacteria	3 days	9.39a	9.45a	9.51a	0.0796	0.5857	***		
	10 days	8.71b	8.85b	8.64b	0.1030	0.3807			
	30 days	8.12c	8.14c	8.18c	0.0729	0.8331			
	60 days	7.19d	6.91d	7.25d	0.1089	0.1248			
	SEM	0.0969	0.0876	0.0923					
	*P* value	<0.0001	<0.0001	<0.0001					

Values with different lowercase letters (a, b, c, and d) indicate significant differences among 3, 10, 30, and 60 days after ensiling of each treatment. Values with different uppercase letters (A, B, and C) indicate significant differences among treatments on the same time during the stable phase. ND, no detected. CK, whole-plant corn silage without any lactic acid bacterial suspensions; CSL, whole-plant corn silage treated with lactic acid bacterial suspensions prepared from whole-plant corn silage; ESL, whole-plant corn silage treated with lactic acid bacterial suspensions prepared from *Elymus sibiricus* silage. SEM, standard error of means. **, P < 0.01; ***, P < 0.001.

**FIGURE 6:**
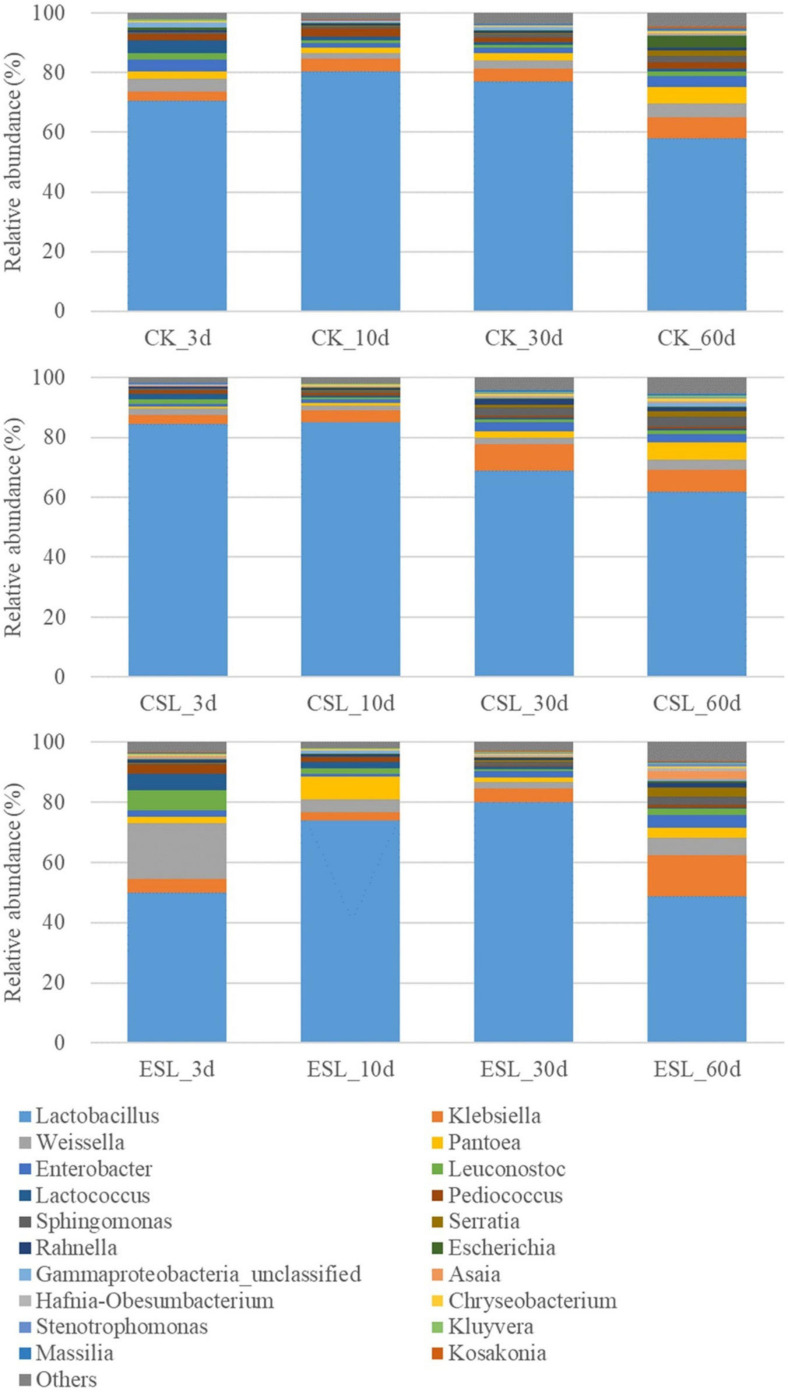
Relative abundance of bacterial communities (genus level) in whole-plant corn silages during the stable phase. CK, whole-plant corn silage without any lactic acid bacterial suspensions; CSL, whole-plant corn silage treated with lactic acid bacterial suspensions prepared from whole-plant corn silage; ESL, whole-plant corn silage treated with lactic acid bacterial suspensions prepared from *Elymus sibiricus* silage.

During this phase, the abundance of *Lactobacillus* reached 80.5% in CK and 85.1% in CSL at 10 days, and 80.1% in ESL at 30 days, and then reduced to 57.9, 61.8, and 48.7% at 60 days, respectively. The opposite trends were observed in *Weissella* (reduced to 1.81 and 1.26% in CK and CSL at 10 days and 2.19% in ESL at 30 days, respectively) and *Leuconostoc* (reduced to 0.83 and 0.66% in CK and CSL at 10 days and 0.86% in ESL at 30 days, respectively). *Lactococcus* in CK, CSL, and ES decreased to 0.85, 0.51, and 0.52% at 60 days, respectively. *Pediococcus* went down to 1.57% in CK and to 0.49% in ESL at 30 days and then up to 2.04 and 0.92% at 60 days, respectively, and that in CSL decreased to 0.57% at 60 days (Figure 6).

### Correlations Between Main Bacterial Genera and pH

**FIGURE 7:**
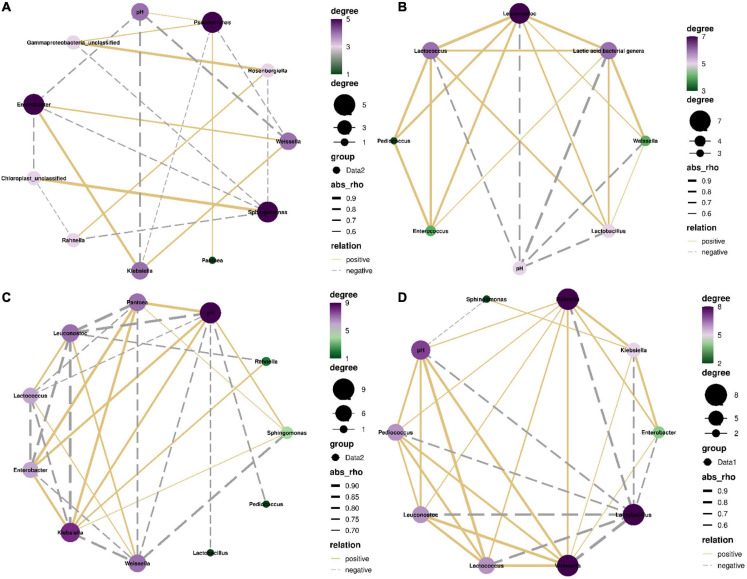
Correlation networks among main bacterial genera (top 10) and pH in the first 5 h **(A)**, from 5 to 24 h **(C)**, and from 24 h to 60 days **(D)**, and among main lactic acid bacterial genera and pH in the first 5 h **(B)**. Absolute value of correlation coefficient >0.5 and *P* value < 0.05.

From 0 to 5 h, the most dominant bacterial genus was *Pantoea* in silages with LAB population as minor taxa (Figures 3, 5). The pH had negative correlations with *Weissella*, *Leuconostoc*, *Lactococcus*, *Pediococcus*, *Lactobacillus*, *Klebsiella*, and *Enterobacter* (*P* < 0.05), and positive correlation with *Pseudomonas* (*P* < 0.05). *Klebsiella* correlated negatively with *Pseudomonas* (*P* < 0.05) and positively with *Weissella* and *Enterobacter* (*P* < 0.05). *Pseudomonas* correlated positively with *Pantoea* and unclassified *Gammaproteobacteria* (*P* < 0.05) and had negative correlation with *Weissella* (*P* < 0.05) (Figures 7A,B).

The most dominant bacterial genus was *Weissella* from 5 to 24 h in silages with LAB population increasing rapidly (Figure 5). The pH had negative correlations with *Weissella*, *Lactococcus*, *Leuconostoc*, *Lactobacillus*, and *Pediococcus* (*P* < 0.05), and positive correlations with *Klebsiella*, *Pantoea*, *Enterobacter*, and *Sphingomonas* (*P* < 0.05). *Weissella* correlated positively with *Lactococcus* and *Leuconostoc* (*P* < 0.05) and negatively with *Klebsiella*, *Pantoea*, *Enterobacter*, and *Sphingomonas* (*P* < 0.05) (Figure 7C).

*Lactobacillus* dominated the bacterial community from 24 h to 60 days (Figure 6). The pH had negative correlations with *Lactobacillus* and *Sphingomonas* and positive correlations with *Weissella*, *Lactococcus*, *Leuconostoc*, *Pediococcus*, and *Rahnella* (*P* < 0.05). *Lactobacillus* correlated negatively with *Weissella*, *Klebsiella*, *Lactococcus*, *Pantoea*, *Leuconostoc*, *Enterobacter*, and *Pediococcus* (*P* < 0.05) (Figure 7C).

## Discussion

Whole-plant corn silages for preparing LAB suspension had lower pH and less LAB count than *E. sibiricus* silages; moreover, LAB suspension-CS contained higher pH and lower LAB count than LAB suspension-ES (Table 1). Those suggested that the LAB in LAB suspension-CS might have good acid tolerance, but the poor capacity of producing acid and proliferation. *Lactobacillus* was the most predominant bacterial genus in LAB suspension-CS and LAB suspension-ES (Figure 1). Additionally, LAB suspension-CS contained greater abundances of *Weissella*, *Lachnoclostridium*_5, and *Clostridium*_sensu_stricto_12 than ESL, which might be due to their inhibited activity by lower pH in LAB suspension-ES (Table 1). *Lachnoclostridium*_5 and *Clostridium*_sensu_stricto_12 were also detected in whole-plant corn silage (Keshri et al., 2018) and in mulberry leave silage and stylo silage (He et al., 2020). The optimum condition for *Lachnoclostridium* growth is neutral to alkaline pH and 20–63°C, and the main fermentation product is acetate by fermenting mono- and disaccharides (Yutin and Galperin, 2013). The high moisture and pH (>4.0) in LAB suspension-CS might provide the satisfactory growing condition for *Clostridium* that is an undesirable microorganism that endangered the fermentation quality of silage (Carvalho-Estrada et al., 2020).

The ensiling process was divided into the initial aerobic phase, the intense fermentation phase, the stable phase, and the aerobic feed-out to better understand the main reactions in silage (Weinberg and Muck, 1996). Ávila and Carvalho (2019) reported the main microorganisms in each phase of fermentation process. In the present study, the initial aerobic phase, the intense fermentation phase, and the stable phase were determined by the oxygen content, the increasing LAB count (pH >4.0), and the decreasing LAB count (pH <4.0), respectively.

The LAB suspensions were cultured in MRS broth anaerobically; however, the LAB was suddenly exposed to oxygen after spraying on ensiling materials. Because of the decreased oxygen-scavenging ability, the complete absence of the oxygen-scavenging system, as well as the accumulation of toxic oxygen metabolites (Zhang and Cai, 2014), the LAB counts in CSL and ESL decreased during the initial aerobic phase (Table 2). Nevertheless, the original epiphytic LAB on whole-plant corn might have the greater oxygen-scavenging ability, owing to the constant exposure to air before ensiling, which resulted in an increasing LAB count in CK during this phase (Table 2). The respiration of aerobic microorganisms and plant cells, and the fermentation of facultatively aerobic or anaerobic bacteria occur synchronously in silage during the initial aerobic phase (Ávila and Carvalho, 2019); those cooperating with plant and microbial proteolytic enzymes cause proteolysis and increase of the buffer capacity in silage (Tao et al., 2012). In the present study, *Pseudomonas* presented in silage with considerable abundance (Figure 3) and can produce proteolytic enzymes and result in protein degradation (Scatamburlo et al., 2015; Paludetti et al., 2020). Those might result in an increase of pH during the initial aerobic phase (Table 2). Additionally, the respiration is much greater than the fermentation and the initial aerobic phase might be mainly depending on the aerobic respiration in silages (Ávila and Carvalho, 2019). In the current study, the duration of the initial aerobic phase was 85, 109, and 90 min in CK, CSL, and ESL, respectively (Figure 2), with 34.6, 25.2, and 0.95% of aerobic bacteria, and 28.2, 57.6, and 86.2% of facultatively anaerobic bacteria in materials, respectively (Supplementary Figure 1). The effect of the respiration of plants and microbes on the initial aerobic phase needs further research. After ensiling, the initial microbial activity is mostly aerobic microflora and then inhibited by an anaerobic or/and sufficiently acidic environment in silage (Dunière et al., 2013). In this study, during the initial aerobic phase, the abundances of *Pantoea*, *Klebsiella*, *Rahnella*, *Erwinia*, and *Serratia* increased in CK, CSL, and ESL, as well as *Enterobacter* in CK and CSL (Figures 3, 4). Nevertheless, the LAB population (*Weissella*, *Enterococcus*, *Lactobacillus*, and *Pediococcus*) present in the silages as minor taxa (Figure 3 and Supplementary Figure 2A), and the bacterial counts increased in CK and decreased in CSL and ESL (Table 2). Those implied that *Enterobacteriaceae* and *Erwiniaceae* represented the major aerobic bacterial activity and LABs have weak activity in whole-plant corn silages during the initial aerobic phase.

During the intense fermentation phase, the most dominant bacterial genus was *Pantoea* from A to 3 h or 5 h with pH >5.5, *Weissella* from 10 to 24 h with pH >4.50 and <5.2, and *Lactobacillus* after 2 days with pH ≤4.25, respectively (Figure 5 and Table 3). Those implied that two shifts of the most predominant bacterial genus (from *Pantoea* to *Weissella* and then to *Lactobacillus*) occurred in whole-plant corn silages during this phase. Similarly, Keshri et al. (2018) found that the most dominant bacterial genus in whole-plant corn silages was *Acinetobacter* in the first 3 h, *Weissella* from 5 h to 1 day, and then *Lactobacillus* after ensiling for 2 days. The LAB count increased exponentially from A to 24 h (Table 3); the abundance of *Weissella*, *Lactococcus*, and *Leuconostoc* had increased before 10 h in CK, CSL, and ESL, while the abundance of Lactobacillus in CK reduced in the first 10 h while it decreased in CSL and ESL in the first 5 h after that they increased (Supplementary Figure 3). Moreover, the CSL and ESL contained greater *Lactobacillus* than CK at 10 h, 24 h, and 2 days (Supplementary Figure 2), and lower pH at 10 h, 24 h, 2 days, and 3 days (Table 3). Those suggested that in whole-plant corn silages with LAB suspension, the fermentation process and *Lactobacillus* succession were initiated early, and *Weissella*, *Lactococcus*, and *Leuconostoc* might grow rapidly and reduce pH (<4.5) for stimulating *Lactobacillus* to multiply and dominate bacterial community during the intense fermentation phase. Additionally, compared with CK and ESL, the CSL had higher *Lactobacillus* at 24 h and 3 days (Figure 5 and Supplementary Figure 2) and lower pH at 24 h, 2 days, and 3 days (Table 3). Those indicated that the *Lactobacillus* in the LAB suspension prepared from whole-plant corn silage had greater adaptive capacity and reduced pH more efficiently in whole-plant corn silages than that in the LAB suspension prepared from *E. sibiricus* silage during this phase. Ali et al. (2020) reported that *Pediococcus* from red clover microbiota exhibited better adaption and dominated the bacterial community in red clover silage at 3 days of ensiling; Ohshima et al. (1997) and Wang et al. (2009) showed that LAB in pre-fermented fluid prepared from ensiling material is more effective at improving the fermentation quality than that prepared from other forage sources. The results mentioned above indicated that the homologous LAB might be more familiar with the physicochemical properties of the silage than other sources of LAB and have more efficiency in promoting microbial succession and fermentation of silage during the early stage. In the study, during the intense fermentation phase, the abundances of *Klebsiella*, *Rahnella*, *Enterobacter*, *Serratia*, *Pantoea*, and *Erwinia* decreased or went up and then down in CK, CSL, and ESL (Figure 5); moreover, the coliform count began to reduce after 10 h with pH <5.2 (Table 3). Those genera belonged to *Enterobacteriaceae* or *Erwiniaceae* and might be inhibited in anaerobic and acidic conditions (pH <5.4) (McGarvey et al., 2013). Keshri et al. (2018) reported that in whole-plant corn silages without inoculant, *Klebsiella* decreased and *Serratia* increased from 3 h to 3 days after ensiling; *Acinetobacter* was the most dominant genus at 3 h. However, in the study, the *Pantoea* was the most dominant genus at 3 h (Figure 5).

The acidic and anaerobic conditions of silage are unsuitable for most of the microorganisms (Ávila and Carvalho, 2019) and might lead to a decrease in the counts of LAB, yeast, and bacteria in whole-plant corn silage with pH <4.0 during the stable phase (Table 4). Moreover, Guan et al. (2020) also found the reducing counts of LAB and yeast from 3 to 60 days in whole-plant corn silage. The LAB population dominated the bacterial community during the stable phase and *Lactobacillus* showed the highest abundance among bacterial genera (Figure 6 and Supplementary Figure 3), which is similar to the results of previous studies in whole-plant corn silages (Keshri et al., 2018; Guan et al., 2020; Xu et al., 2020; Zeng et al., 2020). The abundance of *Lactobacillus* increased from 3 to 10 days in CK and CSL, and to 30 days in ESL, and then reduced in the present study (Figure 6). Similar dynamics were detected in whole-plant corn silages by Zeng et al. (2020). However, Keshri et al. (2018) and Xu et al. (2020) reported that the abundance of *Lactobacillus* increased during fermentation and was more than 90% in final silages; additionally, Guan et al. (2020) found that, from 3 days after ensiling, the abundance increased in the silage at 30°C, while it decreased at 45°C. In the study, during this phase, the other LAB genera (*Weissella*, *Leuconostoc*, *Lactococcus*, and *Pediococcus*) present as minor taxa with less than 5% of abundances, except for *Weissella* in ESL_3 days and ESL_60 days and *Leuconostoc* and *Lactococcus* in ESL_3 days (Figure 6). However, Keshri et al. (2018) detected *Weissella* and *Pediococcus* with considerable abundance from 2 to 30 days, and Guan et al. (2020) reported *Pediococcus* as one dominant bacterial genus from 3 to 14 days at 30°C. The different dynamics of bacterial community among those studies mentioned above might be due to the difference in the corn species, geographical location, mowing period, epiphytic microflora, storing temperature, and inoculants (McGarvey et al., 2013; Guan et al., 2020).

In the first 5 h after ensiling, *Enterobacteriaceae* and *Erwiniaceae* dominated bacterial community in whole-plant corm silage with LAB population as minor taxa pH >5.5 (Supplementary Figure 4 and Tables 2, 3). The abundance of *Klebsiella* and *Enterobacter* increased (Figures 3, 5) because *Enterobacteriaceae* thrived in anaerobic and weak acidic condition (pH >5.4) (McGarvey et al., 2013). Additionally, *Enterobacteriaceae* could ferment water-soluble carbohydrates and lactic acid to acetic acid or other products in silage (Ni et al., 2017). Those might explain the correlation of *Klebsiella* and *Enterobacter* with pH and acetic acid (Figure 7A). The *Weissella*, *Lactococcus*, *Leuconostoc*, and *Lactobacillus* had negative correlation with pH (Figures 7A,B) and their abundances went up during this time (Supplementary Table 1). Nevertheless, the LAB population is minor taxa in the first 3 h and the abundance of *Lactobacillus* in LAB population decreased in the first 5 h (Supplementary Figure 3B). Those indicated that the activity of *Weissella*, *Lactococcus*, and *Leuconostoc* might be the main reason for reducing the pH in whole-plant corn silage in the first 5 h. From 5 to 24 h, the LAB population had a rapidly increasing abundance and dominated the bacterial community after 10 h (Figure 5 and Supplementary Figure 3). *Weissella*, *Lactococcus*, *Leuconostoc*, *Lactobacillus*, and *Pediococcus* had negative correlation with pH (Figure 7C). Additionally, *Weissella* had greater abundance than other LAB genera (Figure 5 and Supplementary Figure 3). Those suggested that the reducing pH of whole-plant corn silage was due to the activity of *Weissella* cooperating with *Lactococcus*, *Leuconostoc*, *Lactobacillus*, and *Pediococcus* during this time. *Klebsiella*, *Pantoea*, *Enterobacter*, and *Sphingomonas* had a reducing abundance and a positive correlation with pH from 5 to 24 h (Figures 5, 7C), indicating that their activity might be inhibited under the acidic condition (pH <5.4). After 24 h, the pH had negative correlation with *Lactobacillus* dominating the bacterial community and correlated positively with *Weissella*, *Lactococcus*, *Leuconostoc*, *Pediococcus*, and *Pantoea* (Figures 5, 6, 7D and Supplementary Figure 3). Those suggested that the activity of *Lactobacillus* caused the reducing pH, and the other LAB genera and *Pantoea* might be restrained effectively by the acidic environment (pH <4.2). Moreover, Xu et al. (2020) also detected a negative correlation between pH and *Lactobacillus* sp. in whole-plant corn silage. Based on the above analysis between the pH and LAB population, the LAB fermentation relay occurred from *Weissella*, *Lactococcus*, and *Leuconostoc* (LAB genera as minor taxa) to *Weissella*, *Lactococcus*, *Leuconostoc*, *Lactobacillus*, and *Pediococcus* (LAB genera rising rapidly), and then to *Lactobacillus* (LAB genera dominating).

## Conclusion

In conclusion, *Lactobacillus*, *Weissella*, and *Lachnoclostridium*_5 were the predominant bacterial genera in LAB suspension-CS; *Lactobacillus* dominated bacterial community in LAB suspension-ES. During the initial aerobic phase, the pH and the abundances of *Pantoea*, *Klebsiella*, *Rahnella*, *Erwinia*, and *Serratia* increased. During the intense fermentation phase, the pH decreased rapidly and the microbial counts increased exponentially; the most predominant bacterial genus shifted from *Pantoea* to *Weissella* and then to *Lactobacillus*; the LAB suspensions promoted microbial succession and LAB suspension-CS was more effective. During the stable phase, the pH and microbial counts decreased, and *Lactobacillus* dominated bacterial community. The LAB fermentation relay occurred from *Weissella*, *Lactococcus*, and *Leuconostoc* to *Weissella*, *Lactococcus*, *Leuconostoc*, *Lactobacillus*, and *Pediococcus*, and then to *Lactobacillus* during the anaerobic fermentation process of whole-plant corn silage.

## Data Availability Statement

The raw data supporting the conclusions of this article will be made available by the authors, without undue reservation.

## Author Contributions

LS, CB, and YX designed the study and wrote the manuscript. LS, CB, HX, NN, YJ, GY, and SL performed the experiments. HX and YJ reviewed and edited the manuscript. LS, CB, and YX analyzed the data. YX funded and supervised the experiments. All authors reviewed the manuscript.

## Conflict of Interest

The authors declare that the research was conducted in the absence of any commercial or financial relationships that could be construed as a potential conflict of interest.

## Supplementary Material

Supplementary Figure 1 Relative abundances of aerobic **(A)** and facultatively anaerobic **(B)** bacteria in whole-plant corn silages. CK, whole-plant corn silage without any lactic acid bacterial suspensions; CSL, whole-plant corn silage treated with lactic acid bacterial suspensions prepared from whole-plant corn silage; ESL, whole-plant corn silage treated with lactic acid bacterial suspensions prepared from *Elymus sibiricus* silage.

Supplementary Figure 2 Difference in bacterial communities (genus level) among treatments at 3 h, 5 h, 10 h, 24 h, 2 day, and 3 day. CK, whole-plant corn silage without any lactic acid bacterial suspensions; CSL, whole-plant corn silage treated with lactic acid bacterial suspensions prepared from whole-plant corn silage; ESL, whole-plant corn silage treated with lactic acid bacterial suspensions prepared from *Elymus sibiricus* silage.

Supplementary Figure 3 Relative abundances of main lactic acid bacterial genera in bacterial community **(A)** and in lactic acid bacterial population **(B)** of whole-plant corn silages. CK, whole-plant corn silage without any lactic acid bacterial suspensions; CSL, whole-plant corn silage treated with lactic acid bacterial suspensions prepared from whole-plant corn silage; ESL, whole-plant corn silage treated with lactic acid bacterial suspensions prepared from *Elymus sibiricus* silage.

Supplementary Figure 4 Relative abundance of bacterial communities (family level) in whole-plant corn silages. CK, whole-plant corn silage without any lactic acid bacterial suspensions; CSL, whole-plant corn silage treated with lactic acid bacterial suspensions prepared from whole-plant corn silage; ESL, whole-plant corn silage treated with lactic acid bacterial suspensions prepared from *Elymus sibiricus* silage.

Supplementary Table 1 Relative abundances (%) of main lactic acid bacterial genera in whole-plant corn silages in the first 5 h.

## References

[B1] Ali N. Wang S. Zhao J. Dong Z. Li J. Nazar M. (2020). Microbial diversity and fermentation profile of red clover silage inoculated with reconstituted indigenous and exogenous epiphytic microbiota. *Bioresour. Technol.* 314:123606. 10.1016/j.biortech.2020.123606 32629380

[B2] Ávila C. L. S. Carvalho B. F. (2019). Silage fermentation—updates focusing on the performance of micro-organisms. *J. Appl. Microbiol.* 128 966–984. 10.1111/jam.14450 31519038

[B3] Cai Y. (1999). Identification and characterization of *Enterococcus* species isolated from forage crops and their influence on silage fermentation. *J. Dairy Sci.* 82 2466–2471. 10.3168/jds.S0022-0302(99)75498-6 10575614

[B4] Carvalho-Estrada P. A. Fernandes J. Silva ÉB. Tizioto P. Paziani S. F. Duarte A. P. (2020). Effects of hybrid, kernel maturity, and storage period on the bacterial community in high-moisture and rehydrated corn grain silages. *Syst. Appl. Microbiol.* 43:126131. 10.1016/j.syapm.2020.126131 32866836

[B5] Denek N. Can A. Avci M. Aksu T. Durmaz H. (2011). The effect of molasses-based pre-fermented juice on the fermentation quality of first-cut lucerne silage. *Grass Forage Sci.* 66 243–250. 10.1111/j.1365-2494.2011.00783.x

[B6] Dunière L. Sindou J. Chaucheyras-Durand F. Chevallier I. Thévenot-Sergentet D. (2013). Silage processing and strategies to prevent persistence of undesirable microorganisms. *Anim. Feed Sci. Tech.* 182 1–15. 10.1016/j.anifeedsci.2013.04.006

[B7] Gharechahi J. Kharazian Z. A. Sarikhan S. Jouzani G. S. Aghdasi M. Salekdeh G. H. (2017). The dynamics of the bacterial communities developed in maize silage. *Microb. Biotechnol.* 10 1663–1676. 10.1111/1751-7915.12751 28696065 PMC5658587

[B8] Guan H. Shuai Y. Yan Y. Ran Q. Wang X. Li D. (2020). Microbial community and fermentation dynamics of corn silage prepared with heat-resistant lactic acid bacteria in a hot environment. *Microorganisms* 8:719. 10.3390/microorganisms8050719 32408707 PMC7285033

[B9] Guan H. Yan Y. Li X. Li X. Shuai Y. Feng G. (2018). Microbial communities and natural fermentation of corn silages prepared with farm bunker-silo in Southwest China. *Bioresour. Technol.* 265 282–290. 10.1016/j.biortech.2018.06.018 29908496

[B10] He L. Chen N. Lv H. Wang C. Zhou W. Chen X. (2020). Gallic acid influencing fermentation quality, nitrogen distribution and bacterial community of high-moisture mulberry leaves and stylo silage. *Bioresour. Technol.* 295:122255. 10.1016/j.biortech.2019.122255 31639626

[B11] Keshri J. Chen Y. Pinto R. Kroupitski Y. Weinberg Z. G. Sela S. (2018). Microbiome dynamics during ensiling of corn with and without *Lactobacillus plantarum* inoculant. *Appl. Microbiol. Biot.* 102 4025–4037. 10.1007/s00253-018-8903-y 29536147

[B12] Khan N. A. Yu P. Ali M. Cone J. W. Hendriks W. H. (2015). Nutritive value of maize silage in relation to dairy cow performance and milk quality. *J. Sci. Food Agr.* 95 238–252. 10.1002/jsfa.6703 24752455

[B13] Klebesadel L. J. (1969). Siberian wildrye (*Elymus sibiricus* L.): agronomic characteristics of a potentially valuable forage and conservation grass for the North. *Agron. J.* 61 855–859. 10.2134/agronj1969.00021962006100060008x

[B14] Li P. You M. Shen Y. (2016). Effects of maturity stage and lactic acid bacteria on the fermentation quality and aerobic stability of Siberian wildrye silage. *Food Sci. Nutr.* 4 664–670. 10.1002/fsn3.312 27625768 PMC5011372

[B15] Logue J. B. Stedmon C. A. Kellerman A. M. Nielsen N. J. Andersson A. F. (2016). Experimental insights into the importance of aquatic bacterial community composition to the degradation of dissolved organic matter. *ISME J.* 10 533–545. 10.1038/ismej.2015.131 26296065 PMC4817675

[B16] McGarvey J. A. Franco R. B. Palumbo J. D. Hnasko R. Stanker L. Mitloehner F. M. (2013). Bacterial population dynamics during the ensiling of *Medicago sativa* (alfalfa) and subsequent exposure to air. *J. Appl. Microbiol.* 114 1661–1670. 10.1111/jam.12179 23521112

[B17] Muck R. (2013). Recent advances in silage microbiology. *Agric. Food Sci.* 22 3–15. 10.23986/afsci.6718

[B18] Ni K. K. Wang F. F. Zhu B. G. Yang J. X. Zhou G. A. Pan Y. (2017). Effects of lactic acid bacteria and molasses additives on the microbial community and fermentation quality of soybean silage. *Bioresour. Technol.* 238 706–715. 10.1016/j.biortech.2017.04.055 28501002

[B19] Ohshima M. Ohshima Y. Kimura E. Yokota H. (1997). Fermentation quality of alfalfa and Italian Ryegrass silages treated with previously fermented juices prepared from both the herbages. *Anim. Feed Sci. Technol.* 68 41–44. 10.2508/chikusan.68.41

[B20] Paludetti L. F. O’Callaghan T. F. Sheehan J. J. Gleeson D. Kelly A. L. (2020). Effect of *Pseudomonas* fluorescens proteases on the quality of Cheddar cheese. *J. Dairy Sci.* 103 7865–7878. 10.3168/jds.2019-18043 32600766

[B21] Romero J. J. Joo Y. Park J. Tiezzi F. Gutierrez-Rodriguez E. Castillo M. S. (2018). Bacterial and fungal communities, fermentation, and aerobic stability of conventional hybrids and brown midrib hybrids ensiled at low moisture with or without a homo- and heterofermentative inoculant. *J. Dairy Sci.* 101 3057–3076. 10.3168/jds.2017-13754 29395147

[B22] Scatamburlo T. M. Yamazi A. K. Cavicchioli V. Q. Pieri F. A. Nero L. A. (2015). Spoilage potential of *Pseudomonas* species isolated from goat milk. *J. Dairy Sci.* 98 759–764. 10.3168/jds.2014-8747 25497792

[B23] Sun Y. M. Zhang N. N. Wang E. T. Yuan H. L. Yang J. S. Chen W. X. (2009). Influence of intercropping and intercropping plus rhizobial inoculation on microbial activity and community composition in rhizosphere of alfalfa (*Medicago sativa* L.) and Siberian wildrye (*Elymus sibiricus* L.). *FEMS Microbiol. Ecol.* 70 218–226. 10.1111/j.1574-6941.2009.00752.x 19702874

[B24] Tao L. Guo X. S. Zhou H. Uundersander D. J. Nnandety A. (2012). Characteristics of proteolytic activities of endo- and exo-peptidases in alfalfa herbage and their implications for proteolysis in silage. *J. Dairy Sci.* 95 4591–4595. 10.3168/jds.2012-5383 22818473

[B25] Wang J. Wang J. Q. Zhou H. Feng T. (2009). Effects of addition of previously fermented juice prepared from alfalfa on fermentation quality and protein degradation of alfalfa silage. *Anim. Feed Sci. Technol.* 151 280–290. 10.1016/j.anifeedsci.2009.03.001

[B26] Wang M. Hou L. Zhang Q. Yu X. Zhao L. Lu J. (2017). Influence of row spacing and p and n applications on seed yield components and seed yield of Siberian wildrye (*Elymus sibiricus* L.). *Crop Sci.* 57 2205–2212. 10.2135/cropsci2016.08.0713

[B27] Weinberg Z. G. Muck R. (1996). New trends and opportunities in the development and use of inoculants for silage. *FEMS Microbiol. Rev.* 19 53–68. 10.1016/0168-6445(96)00025-3

[B28] Xu D. Ding W. Ke W. Li F. Zhang P. Guo X. (2019). Modulation of metabolome and bacterial community in whole crop corn silage by inoculating homofermentative *Lactobacillus plantarum* and heterofermentative *Lactobacillus buchneri*. *Front. Microbiol.* 9:3299. 10.3389/fmicb.2018.03299 30728817 PMC6352740

[B29] Xu D. Wang N. Rinne M. Ke W. Weinberg Z. G. Da M. (2020). The bacterial community and metabolome dynamics and their interactions modulate fermentation process of whole crop corn silage prepared with or without inoculants. *Microb. Biotechnol.* 14 561–576. 10.1111/1751-7915.13623 32627363 PMC7936295

[B30] Yutin N. Galperin M. Y. (2013). A genomic update on clostridial phylogeny: gram-negative spore formers and other misplaced clostridia. *Environ. Microbiol.* 15 2631–2641. 10.1111/1462-2920.12173 23834245 PMC4056668

[B31] Zeng T. Li X. Guan H. Yang W. Liu W. Liu J. (2020). Dynamic microbial diversity and fermentation quality of the mixed silage of corn and soybean grown in strip intercropping system. *Bioresour. Technol.* 313:123655. 10.1016/j.biortech.2020.123655 32559709

[B32] Zhang H. Cai Y. (2014). *Lactic Acid Bacteria: Fundamentals and Practice.* Berlin: Springer.

[B33] Zhang L. Zhou X. Gu Q. Liang M. Mu S. Zhou B. (2019). Analysis of the correlation between bacteria and fungi in sugarcane tops silage prior to and after aerobic exposure. *Bioresour. Technol.* 291:121835. 10.1016/j.biortech.2019.121835 31352166

