# Supplementary figures and images for: Succession of Bacterial Community During the Initial Aerobic, Intense Fermentation, and Stable Phases of Whole-Plant Corn Silages Treated With Lactic Acid Bacteria Suspensions Prepared From Other Silages
Source: Front Microbiol. 2021 Mar 26;12:655095. doi: 10.3389/fmicb.2021.655095 (PMC8032959; doi:10.3389/fmicb.2021.655095)

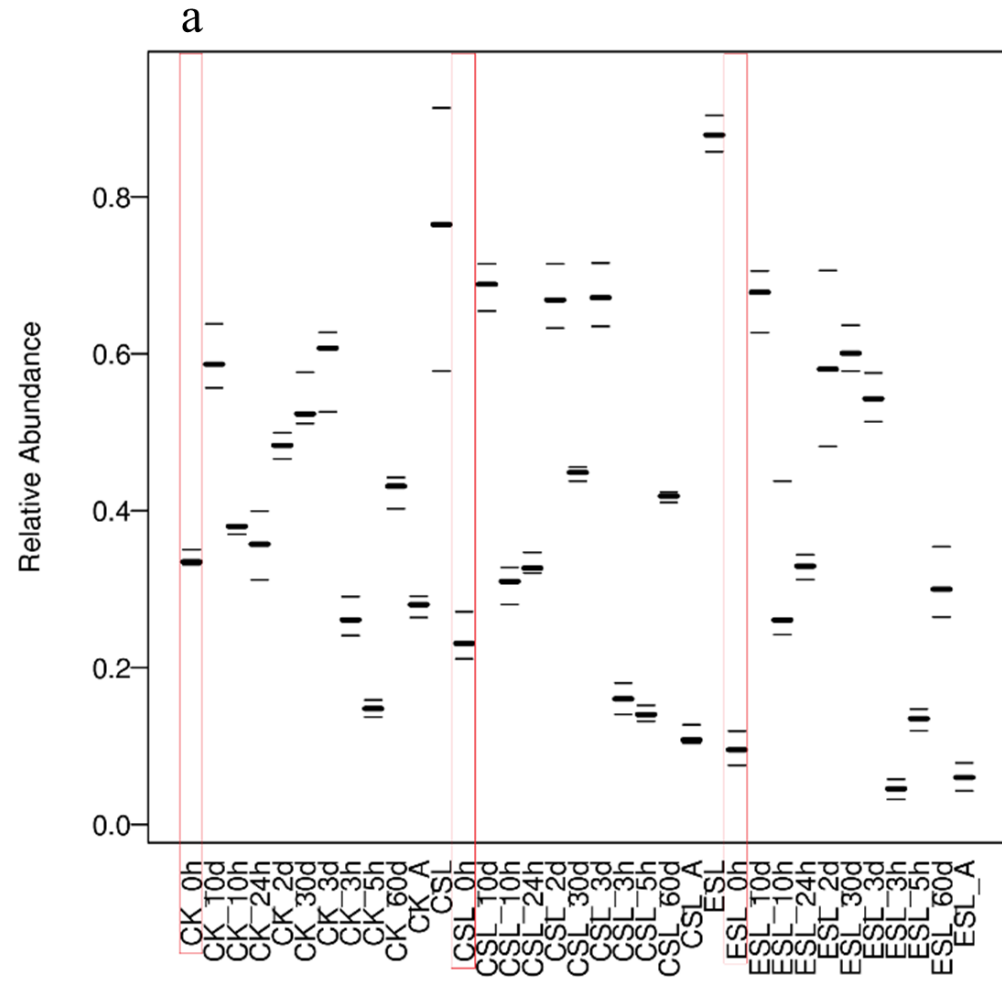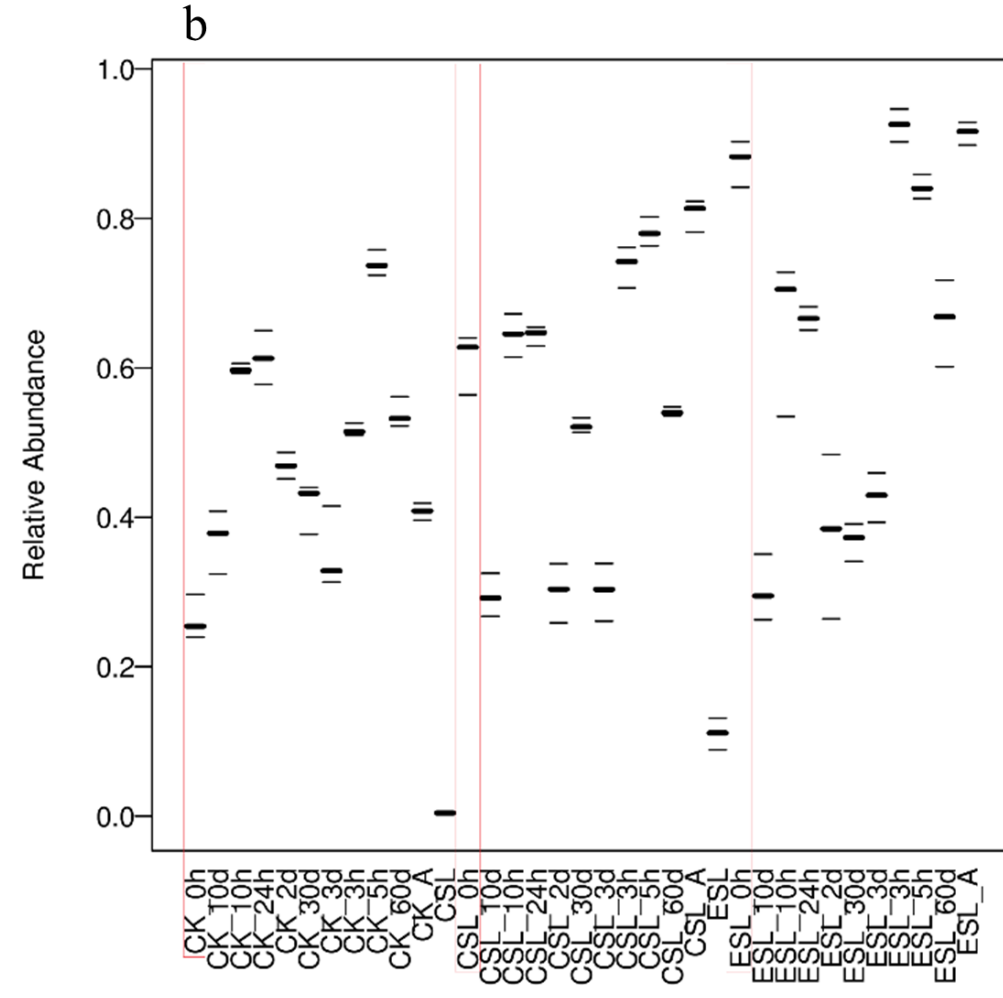

Supplement: Supplementary Figure 1 — Relative abundances of aerobic (A) and facultatively anaerobic (B) bacteria in whole-plant corn silages. CK, whole-plant corn silage without any lactic acid bacterial suspensions; CSL, whole-plant corn silage treated with lactic acid bacterial suspensions prepared from whole-plant corn silage; ESL, whole-plant corn silage treated with lactic acid bacterial suspensions prepared from Elymus sibiricus silage. [file Image_1.pdf]

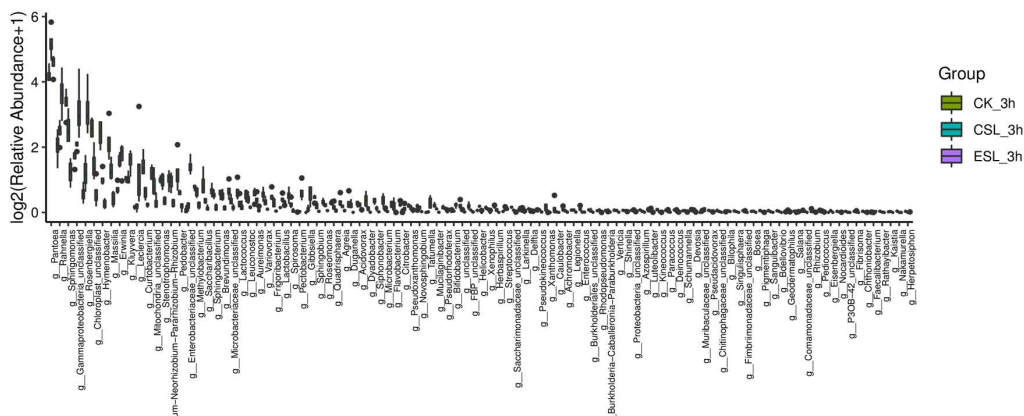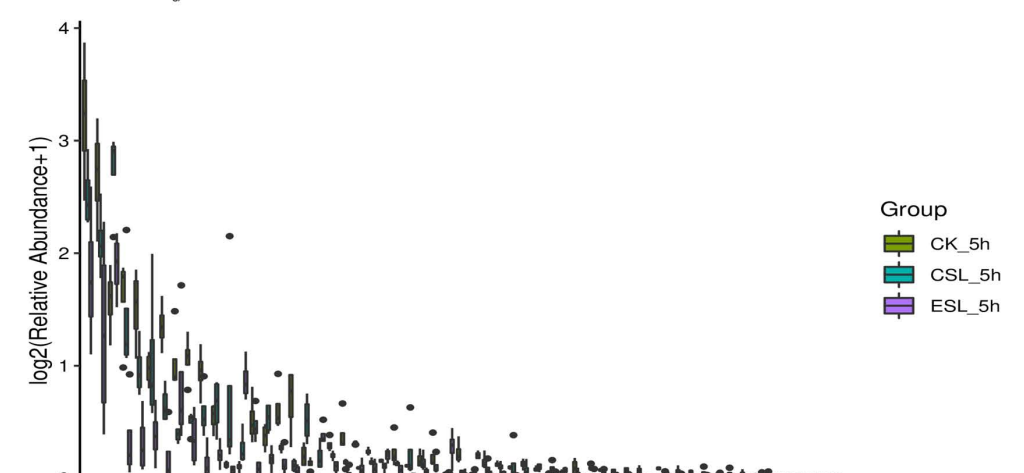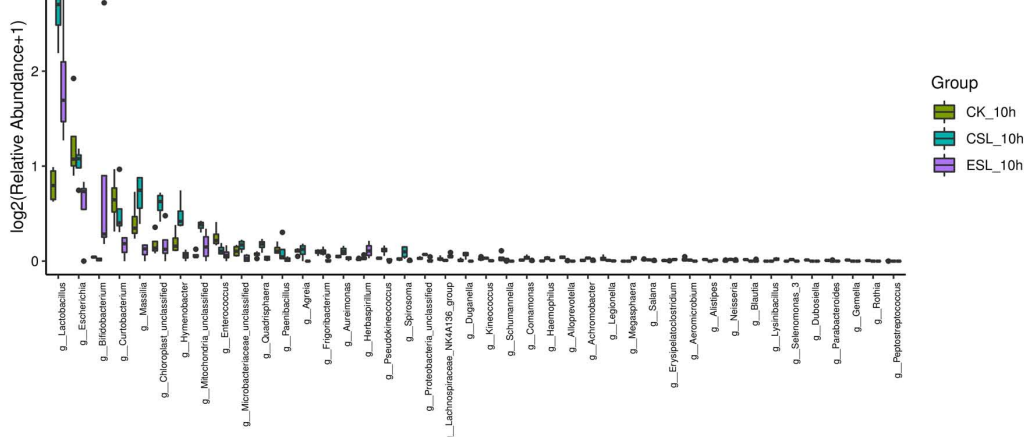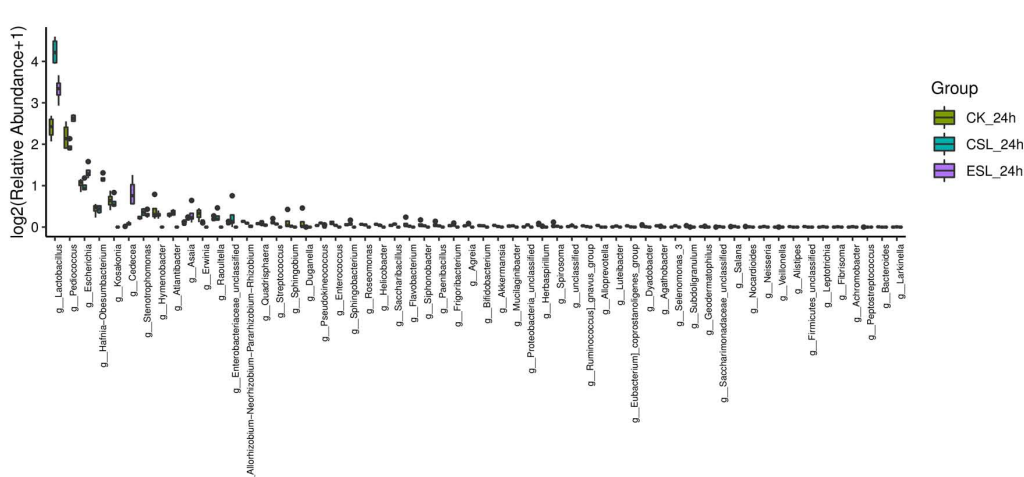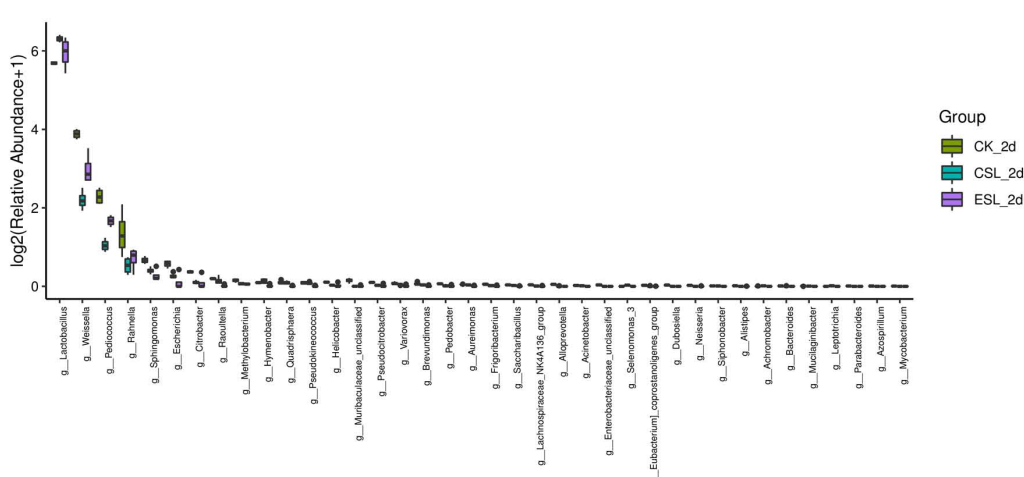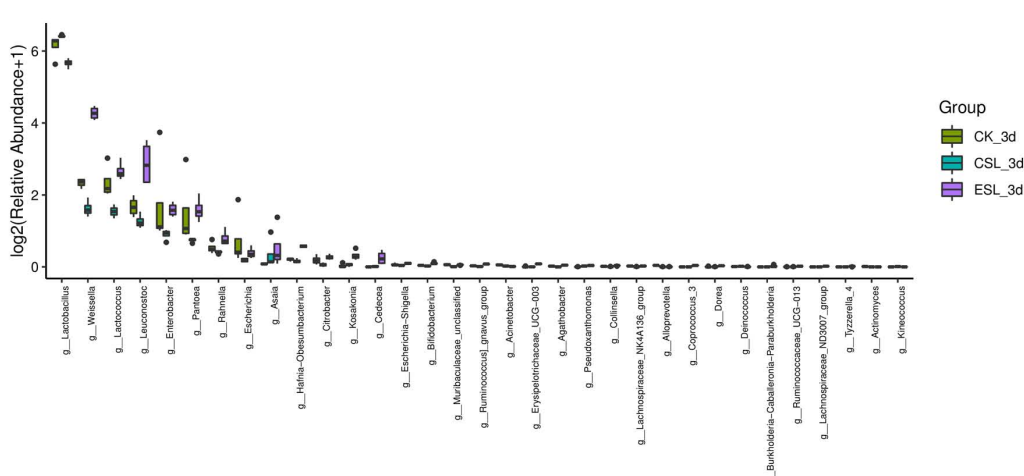

Supplement: Supplementary Figure 2 — Difference in bacterial communities (genus level) among treatments at 3 h, 5 h, 10 h, 24 h, 2 day, and 3 day. CK, whole-plant corn silage without any lactic acid bacterial suspensions; CSL, whole-plant corn silage treated with lactic acid bacterial suspensions prepared from whole-plant corn silage; ESL, whole-plant corn silage treated with lactic acid bacterial suspensions prepared from Elymus sibiricus silage. [file Image_2.pdf]

**a**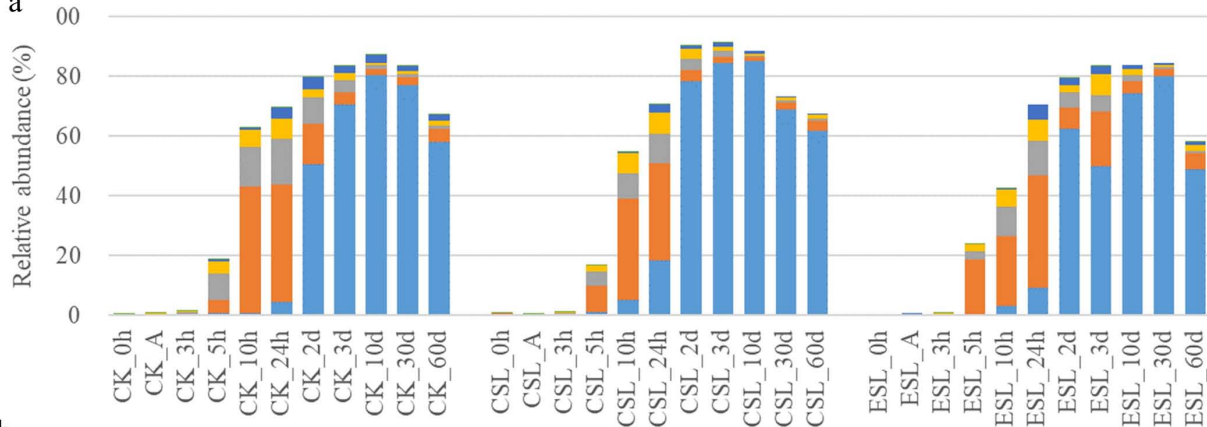**b**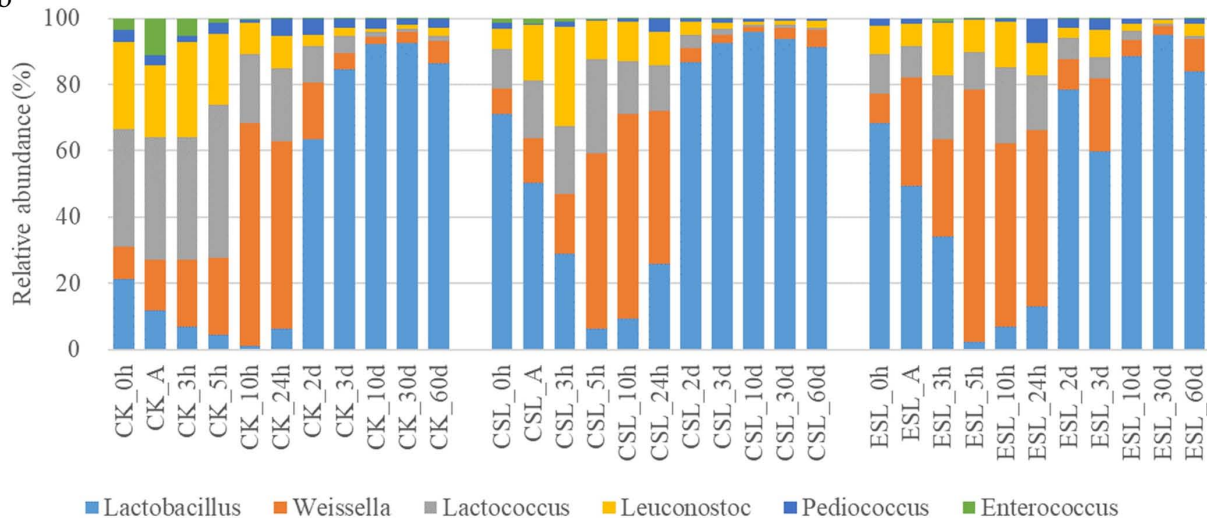

Supplement: Supplementary Figure 3 — Relative abundances of main lactic acid bacterial genera in bacterial community (A) and in lactic acid bacterial population (B) of whole-plant corn silages. CK, whole-plant corn silage without any lactic acid bacterial suspensions; CSL, whole-plant corn silage treated with lactic acid bacterial suspensions prepared from whole-plant corn silage; ESL, whole-plant corn silage treated with lactic acid bacterial suspensions prepared from Elymus sibiricus silage. [file Image_3.pdf]

# Family

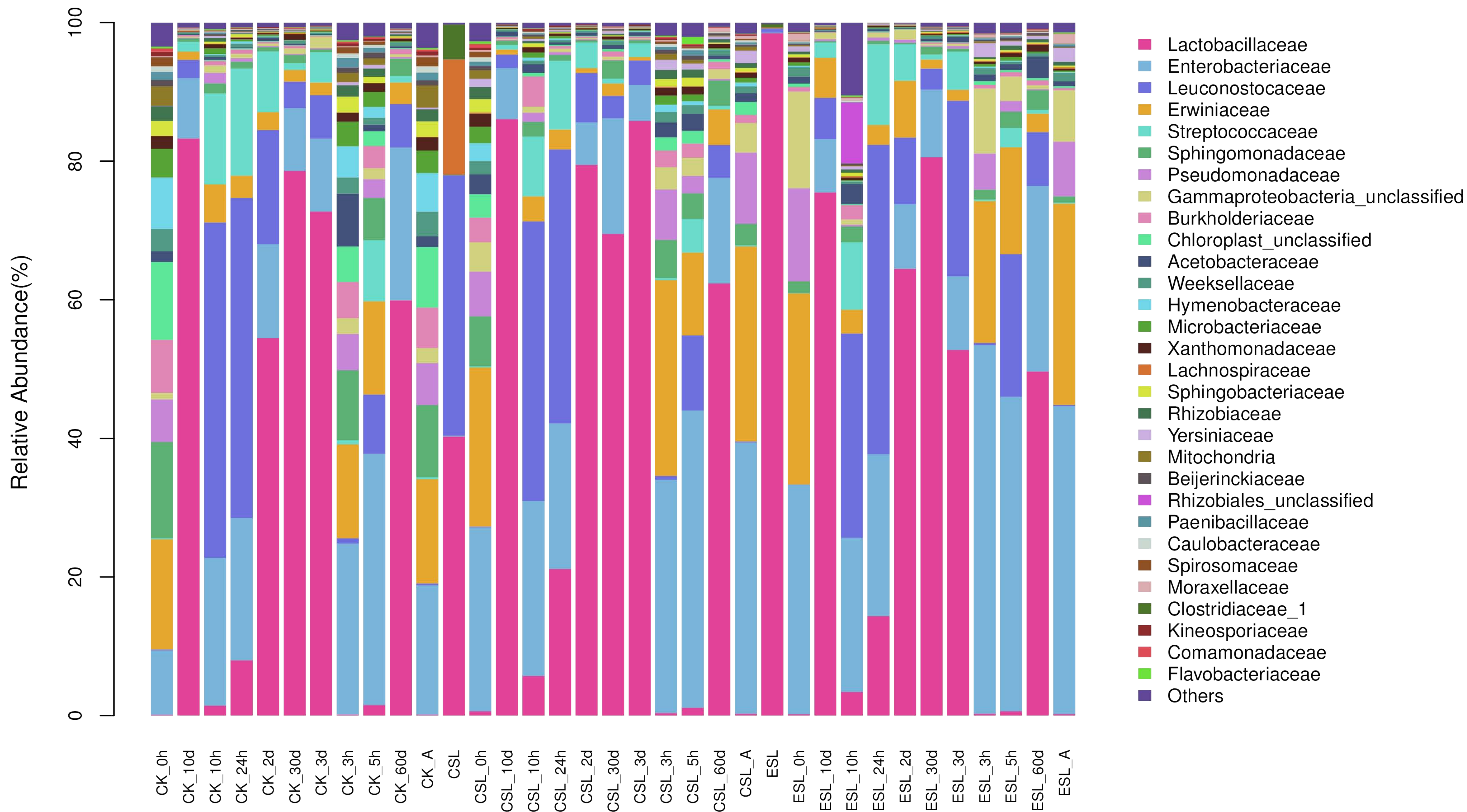

Supplement: Supplementary Figure 4 — Relative abundance of bacterial communities (family level) in whole-plant corn silages. CK, whole-plant corn silage without any lactic acid bacterial suspensions; CSL, whole-plant corn silage treated with lactic acid bacterial suspensions prepared from whole-plant corn silage; ESL, whole-plant corn silage treated with lactic acid bacterial suspensions prepared from Elymus sibiricus silage. [file Image_4.pdf]
