# Supplementary material for: Succession of Bacterial Community During the Initial Aerobic, Intense Fermentation, and Stable Phases of Whole-Plant Corn Silages Treated With Lactic Acid Bacteria Suspensions Prepared From Other Silages
Source: Front Microbiol. 2021 Mar 26;12:655095. doi: 10.3389/fmicb.2021.655095 (PMC8032959; doi:10.3389/fmicb.2021.655095)
Supplement: Supplementary Table 1 — Relative abundances (%) of main lactic acid bacterial genera in whole-plant corn silages in the first 5 h. [file Table_1.docx]

**Table S1** Relative abundances (%) of main lactic acid bacterial genera in whole-plant corn silages in the first 5 hours

| Genus | CK_0h | CK_A | CK_3h | CK_5h | CSL_0h | CSL_A | CSL_3h | CSL_5h | ESL_0h | ESL_A | ESL_3h | ESL_5h |
| --- | --- | --- | --- | --- | --- | --- | --- | --- | --- | --- | --- | --- |
| *Weissella* | 0.040 | 0.109 | 0.321 | 4.451 | 0.065 | 0.066 | 0.209 | 8.906 | 0.024 | 0.133 | 0.208 | 18.266 |
| *Lactococcus* | 0.146 | 0.266 | 0.589 | 8.803 | 0.102 | 0.086 | 0.237 | 4.814 | 0.033 | 0.039 | 0.135 | 2.731 |
| *Leuconostoc* | 0.109 | 0.157 | 0.454 | 4.092 | 0.053 | 0.084 | 0.345 | 1.928 | 0.024 | 0.028 | 0.113 | 2.316 |
| *Lactobacillus* | 0.089 | 0.087 | 0.111 | 0.878 | 0.607 | 0.251 | 0.335 | 1.047 | 0.191 | 0.204 | 0.242 | 0.579 |
| *Pediococcus* | 0.015 | 0.023 | 0.028 | 0.638 | 0.016 | 0.002 | 0.018 | 0.083 | 0.006 | 0.007 | 0.002 | 0.045 |
| *Enterococcus* | 0.015 | 0.080 | 0.086 | 0.246 | 0.011 | 0.008 | 0.012 | 0.067 | 0.000 | 0.000 | 0.008 | 0.061 |

CK, whole-plant corn silage without any lactic acid bacterial suspensions; CSL, whole-plant corn silage treated with lactic acid bacterial suspensions prepared from whole-plant corn silage; ESL, whole-plant corn silage treated with lactic acid bacterial suspensions prepared from *Elymus sibiricus* silage.
